# The FERM guild: a differentially correlated microbial module drives hypertension via metabolic flux perturbations

**DOI:** 10.1128/msystems.00358-26

**Published:** 2026-08-04

**Authors:** Wenkai Lai, Yuchen Zhang, Shaoping Huang, Shirong Lai, Fuxin Lin, Ziwei Wang, Shanwen Sun, Fenglong Yang

**Affiliations:** 1 Department of Bioinformatics, Fujian Key Laboratory of Medical Bioinformatics, School of Medical Technology and Engineering, Fujian Medical University 654110, Fuzhou, China; 2 School of Medical Imaging, Fujian Medical University 74551, Fuzhou, China; 3 Clinical College of Obstetrics and Gynecology and Pediatrics, Fujian Medical University 74551, Fuzhou, China; 4 The First Affiliated Hospital of Fujian Medical University 74551, Fuzhou, China; 5 Biomedical Sciences College & Shandong Medicinal Biotechnology Centre, Shandong First Medical University & Shandong Academy of Medical Sciences 518873, Jinan, China; 6 Key Laboratory of Saline-Alkali Vegetation Ecology Restoration, Ministry of Education (Northeast Forestry University) 47820, Harbin, China; 7 Fujian Key Laboratory of Medical Bioinformatics, Institute of Precision Medicine, Fujian Medical University 74551, Fuzhou, China; 8 Key Laboratory of Gastrointestinal Cancer (Fujian Medical University), Ministry of Education 74551, Fuzhou, China; Florida Atlantic University, Boca Raton, Florida, USA

**Keywords:** hypertension, gut microbial, metabolites, network

## Abstract

Hypertension is a major risk factor for cardiovascular diseases, with changes in gut microbiota composition and function being closely associated with its onset and progression. However, the high inter-individual variability in gut microbiota complicates the identification of pathogenic mechanisms using traditional methods. In contrast, the smaller variability in gut microbial metabolites offers a more reliable and consistent basis for cross-individual comparisons. Parsimonious flux balance analysis (pFBA), integrated with double machine learning (DoubleML), identified 17 metabolites significantly associated with hypertension (*P* < 0.05, robustness value [RV] >0.1). These included meso-2,6-diaminoheptanedioate, p-hydroxyphenylacetic acid, cellobiose, dextran 40 (1,6-α-D-glucan), L-glutamic acid, and kestopentaose, among others. Differential microbial correlation network analysis identified a key microbial subnetwork, termed the FERM guild, consisting of 19 species, with prominent genera including *Faecalibacterium, Enterobacter, Roseburia*, and *Methanobrevibacter*. Using Gene Set Enrichment Analysis (GSEA), the dysregulation of this guild was found to be strongly associated with a set of 17 hypertension-related metabolites (*P* = 0.017). Further analysis revealed that the contribution of FERM genera to key metabolites is significantly associated with blood pressure (*P* < 0.05), even without significant differences in their abundance; additionally, an imbalance exists between FERM genera and other species. Our findings reveal that hypertension is associated with a disruption of gut microbial diversity, structure, and metabolic function. Seventeen key metabolites related to blood pressure regulation were identified, exhibiting pro- or anti-hypertensive potential and linked to functional microbial modules. These results highlight the gut microbiota and its metabolites as promising targets for therapeutic intervention in hypertension.

**IMPORTANCE:**

Hypertension remains a major global public health burden; however, most studies on its relationship with the gut microbiota rely on traditional species-abundance analyses, which are limited by substantial inter-individual variability. In contrast, microbial metabolites show greater stability across individuals and thus offer a more reliable entry point for mechanistic research. By integrating metabolic modeling, causal inference, and network analysis, this study identified 17 key metabolites significantly associated with blood pressure and uncovered a functionally coordinated microbial community (FERM) whose contribution to critical metabolic fluxes (rather than its taxonomic abundance) was closely linked to hypertension. These findings reveal a metabolite-centered mechanism connecting microbial functions to host blood pressure regulation and provide new potential targets for microbiome-based interventions.

## INTRODUCTION

Hypertension remains one of the major risk factors for cardiovascular diseases (CVD) such as stroke and heart failure. In addition, it is an important associated factor for common comorbidities such as chronic kidney disease, obesity, and type 2 diabetes (1, 2). In 2010, approximately 31% of the global population had hypertension, making it a global public health issue (3). Despite extensive research and interventions, blood pressure control still faces many challenges (4).

Recent studies have shown that the gut microbiota plays a crucial role in the development of cardiovascular diseases (5). For instance, patients with heart failure with preserved ejection fraction (HFpEF) exhibit significant gut dysbiosis, with marked differences compared to healthy individuals (6). The gut microbiota metabolizes dietary choline, phosphatidylcholine, and L-carnitine to produce trimethylamine (TMA), which is further oxidized to trimethylamine N-oxide (TMAO), a metabolite known to promote atherosclerosis (5, 7). These findings provide new perspectives and insights into the etiology of hypertension and potential therapeutic strategies.

The gastrointestinal tract is the largest immune cell compartment in the body, representing the intersection between the environment and the host (8). Lifestyle can shape and be influenced by the microbiome (9–11), thereby altering the risk of developing hypertension. A well-studied example is the consumption of dietary fiber, which leads to the production of short-chain fatty acids and contributes to the expansion of anti-inflammatory immune cells, thus preventing the progression of hypertension (12). The gastrointestinal tract is also the primary site for the interactions between dietary components, gut microbiota and their metabolites, and antihypertensive drugs, with blood pressure potentially regulated by gastrointestinal hormones (13).

Although species-level analysis can provide basic information about microbial composition, it does not fully capture the complexity and functional diversity of microbial communities. Species-level analysis overlooks interactions between microorganisms and their functional potential. Currently, research on hypertension-related gut microbiota is relatively limited. Traditional microbial studies often focus on changes in microbial species abundance, such as α-diversity, β-diversity, and species abundance differences (14). However, challenges in microbial research include data sparsity and significant inter-individual variability, which complicate and reduce the accuracy of constructing risk assessment or sample classification models. The interaction networks between microorganisms are crucial for maintaining gut health and metabolic balance (15, 16); however, these interactions may not be fully captured at the species level. The impact of gut microbiota on the host is typically mediated through interactions between their metabolites and the host (17, 18). Compared to simple species analysis, metabolomics has many advantages in deciphering the composition and function of microbes within an organism. Gut microbiota metabolites, as signaling molecules and substrates for host metabolic responses, influence various physiological and pathological processes in the host (19). For example, short-chain fatty acids (SCFAs) are one of the most-studied classes of small molecule metabolites. They are produced by gut microbes through the fermentation of dietary fibers and can regulate host physiological and biochemical functions. These functions include maintaining the natural intestinal barrier at the colonic epithelium and mucus levels, regulating gut motility, secreting gut hormones, modulating chromatin, influencing the gut-brain axis, and supporting immune functions. Metabolites are the end products of metabolic activities in the body, and their composition and abundance directly reflect the physiological state, metabolic pathways, and levels of biological activity within the organism (20, 21). Metabolomics also offers strong biological interpretability. Metabolites are often closely associated with physiological processes and metabolic pathways in the body (22), which endows metabolomic data with significant biological relevance and interpretability.

Recent studies have shown that hypertension is closely related to disturbances in the metabolites of the gut microbiota (23). The gut microbiota can influence host health, particularly through its metabolites. These metabolites can enter the bloodstream and affect the physiological and pathological states of the host. For example, some studies have found that certain gut microbial metabolites, such as SCFAs, can counteract hypertension by modulating immune responses, reducing inflammation levels, regulating energy balance, and cholesterol metabolism (24). Additionally, some gut microbiota-derived metabolites may be involved in blood pressure regulation, such as TMAO produced from dietary nutrients by gut microbes, which has been associated with poor cardiovascular health, including atherosclerosis, hypertension, and heart disease (7).

In this study, parsimonious flux balance analysis (pFBA) was used to calculate the metabolic flux spectrum for each sample. Metabolic flux reflects the rate at which microorganisms produce and consume metabolites, which is related to the state of the host’s disease. This research explores the relationship between metabolites and disease phenotypes, ultimately identifying specific species. We systematically organized microbiome data for comprehensive analysis, constructing a knowledge-based microbial cross-feeding network. This enabled a systematic analysis of the interactions between microbes and metabolites and their relationship with the occurrence of hypertension.

## MATERIALS AND METHODS

### Overview of the study workflow

**Fig 1:**
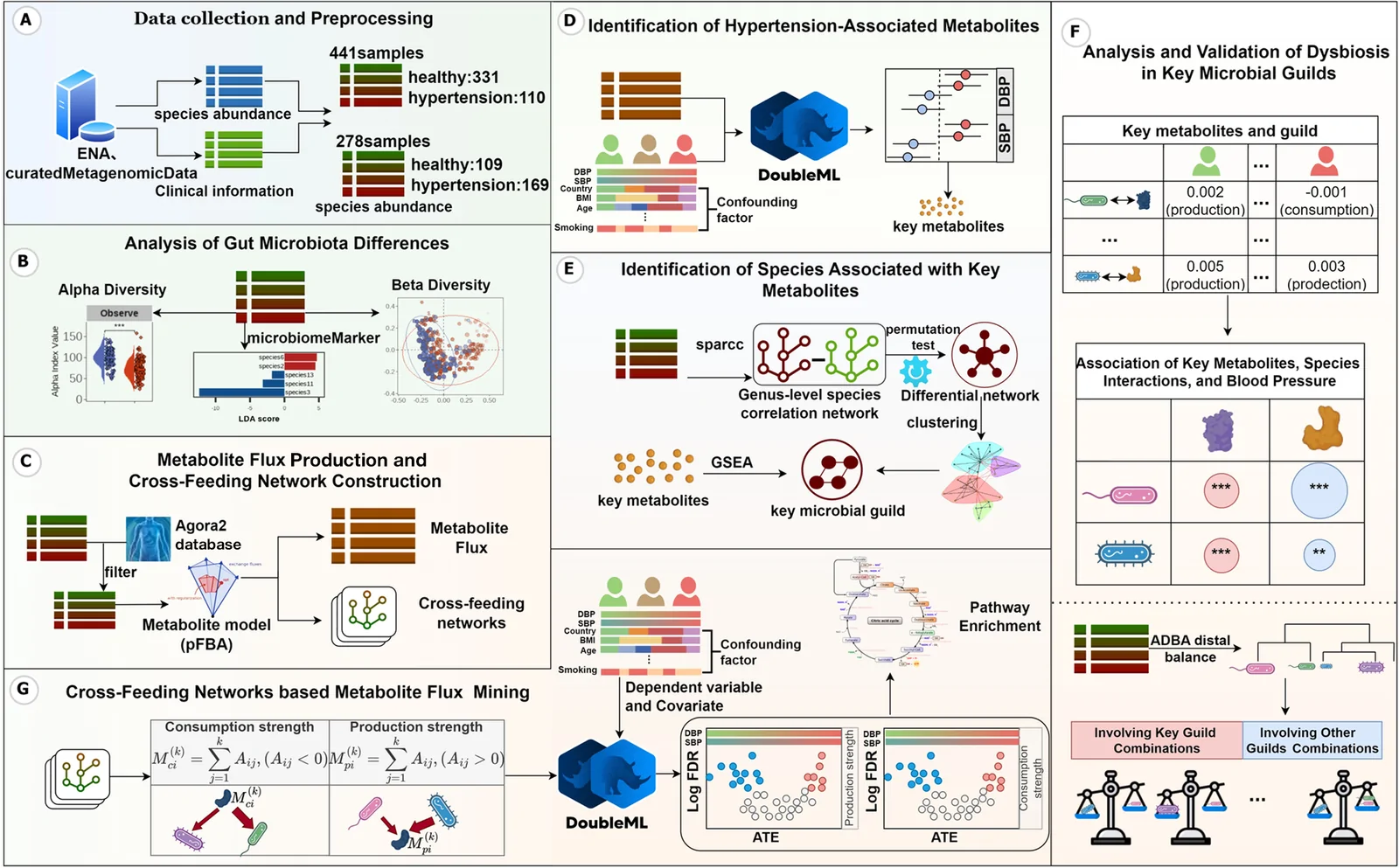
Overview of the research workflow. Downloading and preprocessing of hypertension gut microbiota data (step **A**), analysis of gut microbial differences (step **B**), prediction of metabolic flux based on microbial data (step **C**), identification of hypertension-associated metabolites (step **D**), identification of species associated with key metabolites (step **E**), and analysis and validation of dysbiosis in key metabolites guilds (step **F**). Cross-feeding network-based metabolite flux mining (step **G**)

In this study, 16S rRNA gut microbiome sequencing data related to hypertension, along with corresponding clinical phenotype information, were downloaded from the European Nucleotide Archive (ENA) database. Samples were classified into hypertension and healthy control groups according to the World Health Organization (WHO) blood pressure diagnostic criteria (Fig. 1A). Subsequently, α-diversity and β-diversity analyses were performed, and linear discriminant analysis effect size (LEfSe) was applied to identify microbial taxa with statistically significant differences, with LDA scores calculated to estimate the effect size of each discriminative taxon (Fig. 1B). Using microbial species information recorded in the AGORA2 metabolic model database, samples with low matching fidelity to the database were excluded. The retained samples were then used to infer metabolic fluxes based on pFBA, and a cross-feeding network at the microbial community level was constructed accordingly (Fig. 1C). Integrating clinical blood pressure measurements with medium metabolic flux features (net influx or efflux in the community model), a double machine learning (DoubleML) framework was applied to control for potential confounders. This enabled estimation of the average treatment effects of individual metabolites on systolic and diastolic blood pressure, followed by sensitivity analyses to identify robust candidate metabolites (Fig. 1D). Building on this, gene set enrichment analysis (GSEA) was employed to evaluate the enrichment of key metabolites within distinct microbial clusters (Fig. 1E). To further investigate the dysbiosis of key guilds, this study extracted the key metabolites and species interactions from the cross-feeding network. We performed association analysis between these interactions and blood pressure in both the discovery and validation cohorts. Additionally, we conducted ADBA distal balance analysis on the species within key guilds to validate their dysbiosis (Fig. 1F). Finally, the production and consumption rates of metabolites at the community level were quantified, and their associations with blood pressure phenotypes were analyzed after adjustment for confounders using DoubleML. Metabolic pathway enrichment analysis was further conducted on metabolites showing significant associations to elucidate their potential physiological functions and mechanisms (Fig. 1G).

### Data collection and sample grouping

In this study, species abundance tables derived from 441 fecal microbiota samples (16S rRNA sequencing) were obtained from the work of Jacobo de la Cuesta-Zuluaga et al. (25; available at https://github.com/jsescobar/westernization/tree/master/files). Each sample was accompanied by detailed clinical information, including systolic and diastolic blood pressure, age, gender, body mass index (BMI), cholesterol, low-density lipoprotein (LDL), cardiometabolic disease status, and medication use. According to the World Health Organization (WHO) definition of hypertension (diastolic blood pressure ≥90 mmHg or systolic blood pressure ≥140 mmHg), participants were classified into a hypertension group (*n* = 110) and a healthy control group (*n* = 331) for downstream analyses. Additionally, we collected 169 hypertension samples from curatedMetagenomicData, a specialized database for human gut microbiome data. Since the age distribution of the hypertension samples ranged approximately from 55 to 70 years, and the samples were primarily from Austria (AUT), China (CHN), and Italy (ITA), we established matching criteria for healthy controls to minimize confounding effects. The selection criteria for healthy samples were as follows: age between 55 and 70 years, country of origin limited to AUT, CHN, or ITA, and a BMI between 18.5 and 23.9. Based on these criteria, 109 healthy samples were obtained, resulting in a total of 278 samples. The species abundance data for these samples can be retrieved in R using the corresponding sample names provided in Table S1. This data set was used for validating the statistical analysis results.

### Construction of community metabolic flux models and cross-feeding networks

To simulate gut microbiota interactions, we utilized the AGORA2 database (v2.01), which comprises genome-scale metabolic models (GEMs) for 7,302 microbial strains (26). These models provide detailed stoichiometry and directionality for all known metabolic reactions, curated from genomic sequences, functional annotations, and strain-specific biochemical data. To adapt these strain-specific models for our analysis, we constructed genus-level metabolic models by aggregating the reactions of all constituent strains into unified models while removing redundant reactions. For each genus-level model, the biomass objective function was constructed by averaging the stoichiometric coefficients of the biomass reactions from all strains belonging to the same genus.

Subsequently, genus taxa from 441 samples were matched against this consolidated database. To ensure high-fidelity simulations, we retained only those samples where the matched taxa accounted for more than 80% of the total relative abundance. Given that all samples were collected from Colombia with relatively uniform dietary patterns, a standardized Western diet constraint (27) was applied. Subsequently, we employed the micom framework and incorporated genus-level genome-scale metabolic models from the AGORA2 database to construct microbial community models. Community optimization was performed using parsimonious flux balance analysis (pFBA) (28, 29), with the objective of maximizing community biomass while minimizing total metabolic flux. This approach enabled us to estimate metabolite production and consumption fluxes for each genus. Based on predicted secretion and uptake fluxes, metabolite-mediated interactions between genera were inferred by linking metabolites secreted by one taxon to those consumed by another within the same community context, thereby generating sample-specific cross-feeding networks.

The present framework focused specifically on the metabolic potential of the gut microbiota and therefore did not explicitly incorporate host metabolic reactions. As a result, the inferred metabolite exchanges should be interpreted as microbiome-derived metabolic capacities rather than direct measurements of systemic host metabolite levels.

### Identification of hypertension-associated metabolites

To investigate the relationship between metabolic fluxes and hypertension, we first filtered the metabolites by extracting the community-level metabolites (metabolites representing the overall import and export fluxes between the microbial community and the external medium). We then retained only those metabolites with nonzero predicted fluxes in at least 10% of the samples. Given that the underlying mechanisms leading to elevated diastolic and systolic blood pressure may differ, we conducted separate analyses to identify metabolites associated with diastolic blood pressure (DBP) and systolic blood pressure (SBP). To comprehensively control for the potential influence of confounding factors on the study outcomes, the following variables were included as covariates for adjustment in the model: age, adiponectin levels, BMI, body fat percentage, fasting glucose, glycated hemoglobin (HbA1c), high-density lipoprotein (HDL), high-sensitivity C-reactive protein (hs-CRP), insulin levels, total cholesterol, triglycerides, very low-density lipoprotein (VLDL), waist circumference, residential location (city), gender, smoking status, and medication use.

We applied the DoubleML framework with double random forests (30), a method specifically designed for causal inference. Separate models were constructed for DBP and SBP to estimate the average treatment effect (ATE) of each metabolite. To control for confounding and reduce bias from overfitting, we implemented a 5-fold cross-fitting procedure. Let *X* denote the covariates, *Y* denote the outcome (blood pressure), and *D* denote the treatment (metabolite flux). The conditional expectation functions (nuisance functions) *E*[*Y*|*X*] = *m*(*X*) and *E*[*D*|*X*] = *g*(*X*) were estimated on the training folds, and residuals on the held-out fold were computed as $\tilde{Y}=Y-m(X)$ and $\tilde{D}=D-g(X)$, isolating the variation not explained by confounders. The residualized outcome $\tilde{Y}$ was then regressed on the residualized treatment $\tilde{D}$, and the resulting coefficient θ represents the ATE. Since *D* is continuous, θ can be interpreted as the average marginal effect of the treatment on the outcome. To further verify that our models were not affected by substantial overfitting, we assessed the variability of the estimated ATE across different cross-validation splits. Specifically, we repeated the entire 5-fold cross-fitting procedure 1,000 times, each with a random partitioning of the data. For each repetition, an ATE estimate was obtained, resulting in a distribution of 1,000 ATE values. We then calculated the coefficient of variation (CV) as follows:


(1) $$CV=\frac{sd(ATE)}{|mean(ATE)|}$$

A small CV indicates that the ATE estimates are stable across different cross-validation splits.

To evaluate the potential influence of unobserved confounding, sensitivity analyses were conducted within the DoubleML framework (31). The sensitivity parameters ${cf}_{d}$ and ${cf}_{y}$ were used to quantify the residual associations of hypothetical unobserved confounders with the exposure and the outcome, respectively. Given the complexity of host-microbiome interactions and the possibility that factors such as dietary heterogeneity and host metabolic variation were not fully captured in the available data, residual confounding beyond the adjusted covariates may still exist. Therefore, a moderate residual confounding scenario was evaluated by setting ${cf}_{d}$ = ${cf}_{y}$ = 0.1, corresponding to an unobserved confounder explaining approximately 10% of the remaining variance in both the exposure and the outcome after adjustment for observed covariates. To further quantify robustness beyond this predefined scenario, robustness values (RV) were additionally calculated for each metabolite to estimate how strong residual confounding would need to be to render the estimated treatment effect statistically insignificant. Larger RV values indicate that stronger hidden confounding would be required to explain away the observed association.

Finally, to evaluate the overall characteristics of the cross-feeding subnetwork constructed with key metabolites and their associated species as nodes, we calculated the network density and average shortest path length for this subnetwork in each sample. Based on these metrics, we further compared differences in network density and average shortest path length of the subnetwork between hypertensive patients and healthy controls.

### Network-based mining of network topology characteristics

#### Microbial correlation differential network analysis

Gut microbiota can influence host health through their metabolic products. Given that gut microorganisms often function cooperatively, we calculated pairwise correlations between microbial species using the SparCC method. SparCC (Sparse Correlations for Compositional data) (32) is specifically designed for compositional microbiome data and estimates approximate linear correlations between species by accounting for compositional constraints and data sparsity. To capture condition-specific microbial interactions, correlations were computed separately for the hypertension and healthy groups based on their respective species abundance matrices. The resulting correlation matrices were then sparsified using the overall mean correlation within each group as a threshold, thereby constructing microbial correlation networks for the hypertension group ($G_{hypertension}$) and the healthy group ($G_{healthy}$). Finally, a differential microbial network (ΔG) was obtained by subtracting the edge weights of the healthy network from those of the hypertensive network.


(2) $$\Delta G=G_{hypertension}-G_{healthy}$$

We then performed permutation tests (33) to evaluate the statistical significance of edge-level differences between the healthy and hypertensive networks. For each edge, a permutation-derived *P*-value was calculated, and edges that did not meet the predefined significance threshold were removed from the differential network.

#### Clustering and enrichment of microbial nodes in the differential network related to metabolically important features

To identify microbial communities potentially involved in key metabolic alterations, the differential microbial correlation network was partitioned using a multi-level modularity optimization algorithm (34). Subsequently, the GSEA method (35, 36) was used to evaluate the enrichment of each guild in key metabolic features. Specifically, microbial species were ranked based on the stability of their associations with key

metabolites. The stability metric *Si* was calculated as follows:


(3) $$S_{i}=\sum_{k=1}^{n}\sum_{j}a_{ij}^{\left(k\right)}$$

where $S_{i}$ represents the overall stability of the association between microbial species *i* and the set of key metabolites; *k* denotes the *k*th sample; *j* denotes the *j*th key metabolite; and $a_{ij}^{\left(k\right)}$refers to the element of the binary adjacency matrix in the cross-feeding network in sample *k*.

Subsequently, microbial species were ranked in descending order based on their $S_{i}$ values, and microbial guilds identified from the differential network were used as candidate gene sets for GSEA to identify guilds highly associated with the regulation of key metabolites.

### Analysis and validation of dysbiosis in key microbial guilds

To elucidate the differential roles of the FERM guild in regulating key metabolic fluxes, we first extracted, for each sample, subnetworks from the cross-feeding network in which nodes correspond to FERM guild microbes and key metabolites, thereby defining microbe-metabolite flux relationships.

In the discovery cohort, the DoubleML framework was applied to estimate the ATE of each microbe-metabolite flux on systolic and diastolic blood pressure (SBP and DBP). In this setting, microbe-metabolite fluxes were defined as exposure variables, while blood pressure traits were treated as outcome variables. This analysis followed the same framework used for key metabolite identification, where each microbe-metabolite flux feature was modeled as a treatment variable and its causal effect on blood pressure was estimated while adjusting for potential confounders using machine learning-based nuisance models. The main output of this analysis was the estimated ATE of each microbe-metabolite flux on blood pressure. In the validation cohort, no quantitative blood pressure measurements were available, and only categorical phenotypes (hypertension vs. control) were included. Therefore, causal effect estimation using DoubleML was not applicable. Instead, we performed a group-level validation analysis in which microbe-metabolite fluxes were treated as input features and disease status as the grouping variable. Non-parametric Wilcoxon rank-sum tests were used to assess differences in flux distributions between the two groups. In addition, log fold changes (logFC) were calculated for each microbe-metabolite flux to quantify the direction and magnitude of group-level differences between hypertensive and control subjects. The resulting logFC values were used as descriptive measures of differential microbe-metabolite flux. Finally, we compared the sign of logFC with the sign of the ATE estimates from the discovery cohort to evaluate directional consistency between group-level differences and inferred causal effects.

Furthermore, to comprehensively assess the degree of imbalance within the FERM guild, we applied the ADBA distal balance method (37). This procedure first constructs a sequential binary partition matrix using the sbp.fromADBA function in the R package balance (37), which encodes the partitioning structure of taxa into positive and negative groups and serves as the basis for subsequent log ratio transformations. Each resulting balance *b_i_* was then calculated as a weighted log ratio of the geometric means of taxa in the positive and negative partitions:


(4) $$b_{i}=\sqrt{\frac{|i_{p}|\;|i_{n}|}{|i_{p}|+|i_{n}|}}\log\left(\frac{g(i_{p})}{g(i_{n})}\right)$$

where *g(x*) is the geometric mean of *x*, $i_{p}$ is the sub-composition of positively valanced components, and $i_{n}$ is the sub-composition of negatively valanced components. Here, $i_{p}$ describes the norm, or length, of the sub-composition.

We define an “imbalanced balance” as a balance whose distribution differs significantly between groups (hypertension vs. controls), as assessed using the Wilcoxon rank-sum test. This allows identification of microbial log-ratio structures that are significantly shifted across conditions. Finally, we quantified the number of significantly imbalanced balances associated with FERM taxa to evaluate the overall degree of compositional disruption within this guild.

## RESULTS

### Hypertension-associated alterations in gut microbiota

**Fig 2:**
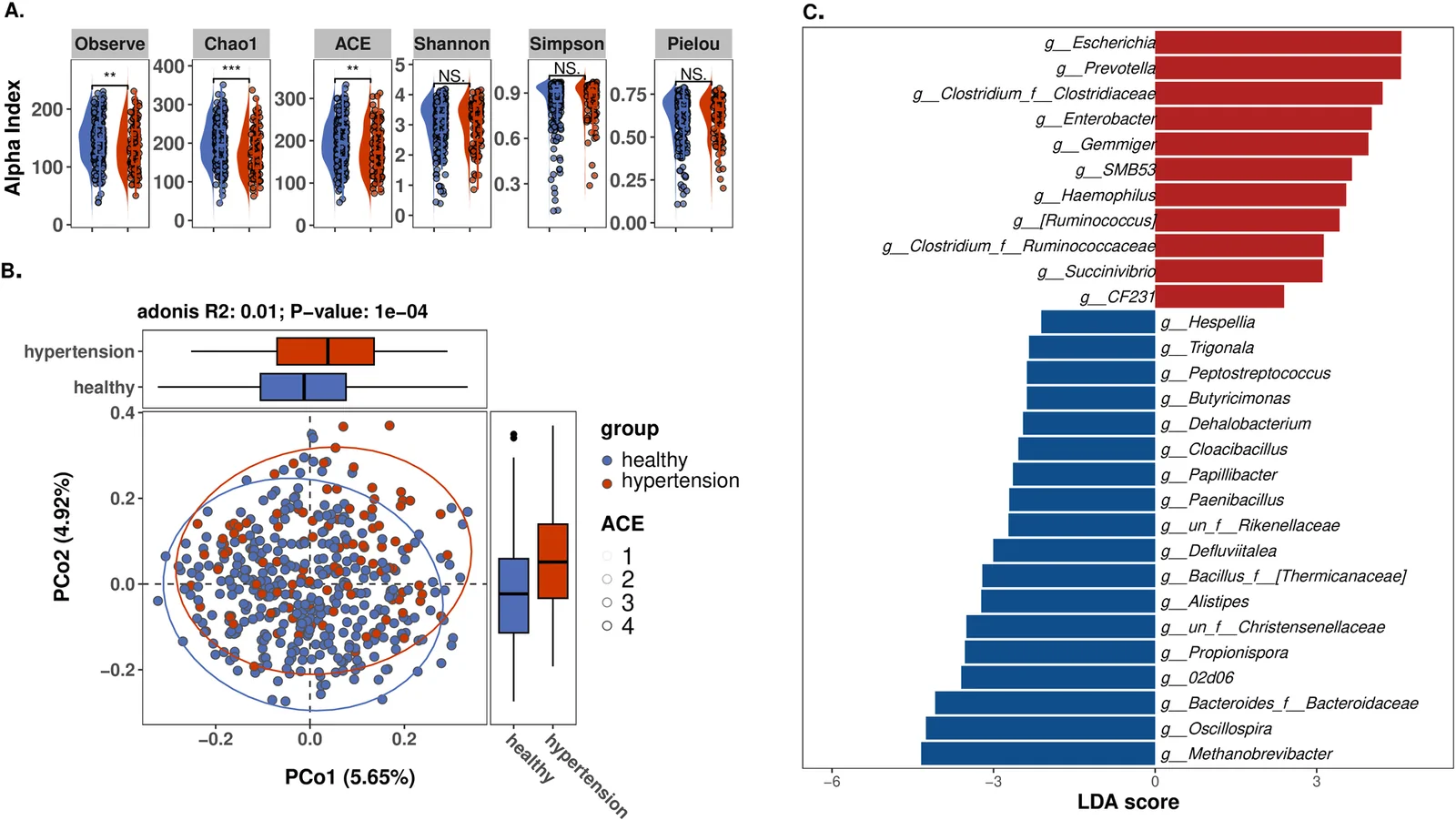
Hypertension-associated alterations in gut microbiota. Wilcoxon rank-sum test revealed significant differences in the observe, Chao1, and ACE α-diversity indices between hypertensive and healthy individuals. Red indicates hypertensive patients, while blue denotes healthy controls (**A**). Significant gut microbial composition differences between the hypertensive and healthy samples (**B**). A larger proportion of gut microbial species exhibit reduced abundance in individuals with hypertension (**C**).

In this study, gut microbial data were divided into two groups based on disease status: hypertensive and healthy. α-Diversity analysis on the gut microbiota of both the hypertensive and healthy groups was conducted, six indices: observe, Chao1, ACE, Shannon, Simpson, and Pielou were calculated. Observe, Chao1, and ACE showed significant differences, with the healthy group exhibiting higher values than the hypertensive group (Fig. 2A), indicating a decrease in microbial richness in hypertensive patients while evenness showed no significant difference.

The β-diversity analysis based on Bray-Curtis distance was conducted to assess differences in gut microbial community structure between the hypertension and healthy groups. The analysis showed that group status explained approximately 1% of the total variance (*R*^2^ = 0.01). The permutation test indicated that the difference was statistically significant (*P* = 10^−4^) (Fig. 2B).

Using LEfSe, we identified a total of 29 genera that showed significant differences between the hypertension and healthy groups, with 11 genera enriched in the hypertension group and 18 genera enriched in the healthy group (Fig. 2C). When extending the LEfSe across all taxonomic levels (kingdom, phylum, class, order, family, genus, and species) without restriction, we detected 64 taxa enriched in the healthy group and 32 taxa significantly enriched in the hypertension group (Fig. S1; Table S2). Overall, these findings indicate that the gut microbial abundance in hypertension patients tends to be reduced, consistent with the decreased species richness observed in the previous α-diversity analysis. However, from an overall perspective, the β-diversity analysis showed that the variance explained by the principal coordinates in the PCoA, as well as the $R^{2}$ value from the PERMANOVA analysis, were both low, indicating limited differences in microbial community structure. Therefore, it is scientifically reasonable and necessary to further investigate the system from the perspective of metabolic flux and microbial interspecies interactions.

### Altered community-level medium metabolite fluxes in the gut microbiota of hypertensive patients

**Fig 3:**
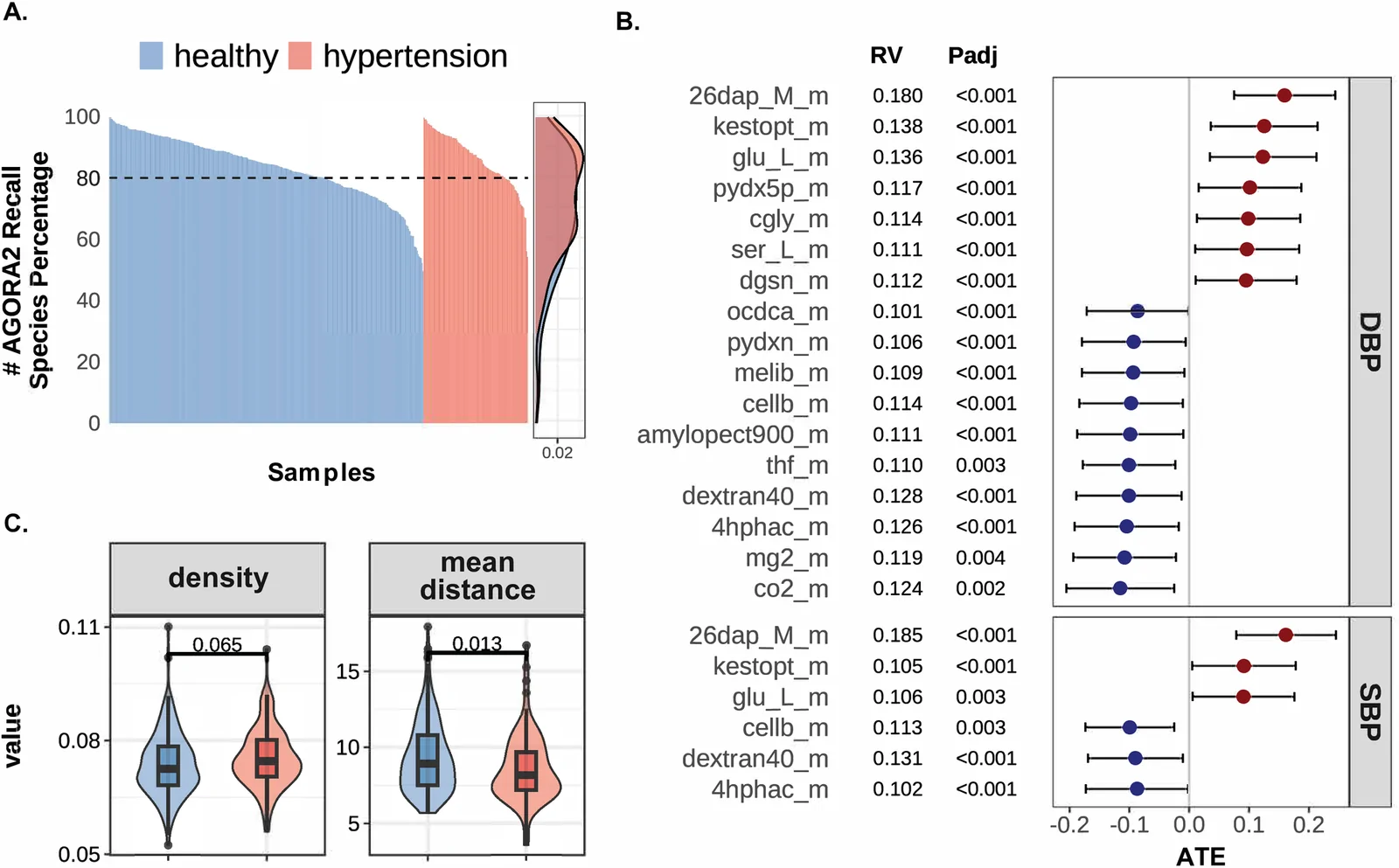
Altered community-level medium metabolite fluxes in the gut microbiota of hypertensive patients. Samples were filtered based on a matching threshold of less than 80% with the AGORA2 database. The height of each bar represents the cumulative relative abundance of microbial species in the sample that successfully matched the AGORA2 reference models. Red bars indicate hypertensive patients, and blue bars represent healthy individuals (**A**). Seventeen metabolites exhibited significant average treatment effects on diastolic blood pressure, while six metabolites showed average treatment effects on systolic blood pressure. Red indicates a positive average treatment effect, and blue indicates a negative average treatment effect. RV represents the robustness value derived from sensitivity analysis (**B**). The average shortest path length of the cross-feeding network for 17 metabolites is significantly reduced in hypertension (**C**).

Analyzing species abundance data alone cannot fully capture the complexity and functional variability of microbial communities, and it may overlook interactions and functional potential among microbes. Therefore, metabolic fluxes were predicted and systematically evaluated to reveal their potential associations with hypertension. To enable accurate modeling of gut microbiota metabolic fluxes, the detected microbial species were mapped to the AGORA2 database. Samples in which the cumulative relative abundance of the mapped species fell below 80% were excluded. Following this filtering step, 312 samples were retained, including 226 from healthy individuals and 86 from patients with hypertension (Fig. 3A). Metabolic flux distributions were inferred using pFBA, allowing the systematic estimation of reaction-level metabolic activity within the microbial community.

Using DoubleML analysis, we identified 17 metabolites significantly associated with DBP, among which seven metabolites showed a positive average treatment effect (ATE > 0) and 10 showed a negative ATE (ATE < 0). Additionally, six metabolites were found to be significantly associated with SBP, with three metabolites having *ATE* >0 and three having ATE <0. Notably, the six systolic blood pressure-associated metabolites completely overlapped with those identified for diastolic blood pressure. Statistical analysis showed that the false discovery rate (FDR) for these metabolites was below 0.005. Sensitivity analyses further yielded robustness values (RV) greater than 0.1 (Fig. 3B; Table S3). In addition, the RMSE values of the exposure and outcome nuisance models from the 5-fold cross-fitting procedure were consistently low across folds (Table S3). To assess the stability of the estimated average treatment effects (ATEs) for each metabolite across different cross-validation splits, we calculated the coefficient of variation (CV). The resulting CVs were 0.147 ± 0.019 for DBP and 0.164 ± 0.033 for SBP, indicating that the ATE estimates were stable across folds (Table S3). These results suggest that the estimated associations were relatively stable and not substantially driven by overfitting or unobserved confounding.

For each sample, cross-feeding networks were constructed, and subnetworks composed of hypertension-associated metabolites were extracted. Network density and average shortest path length were calculated. No significant difference was observed in network density between the hypertension and healthy groups (*P* = 0.065); however, the average shortest path length was significantly lower in the hypertension group compared to the healthy group (*P* = 0.013). These findings suggest that although the number of species contributing to the hypertension-associated metabolites did not significantly differ between groups, the rates at which these metabolites were produced or consumed by the microbial community were significantly elevated in hypertension (Fig. 3C).

### Dysregulation of community-level medium metabolic flux driven by a functional group disturbance

**Fig 4:**
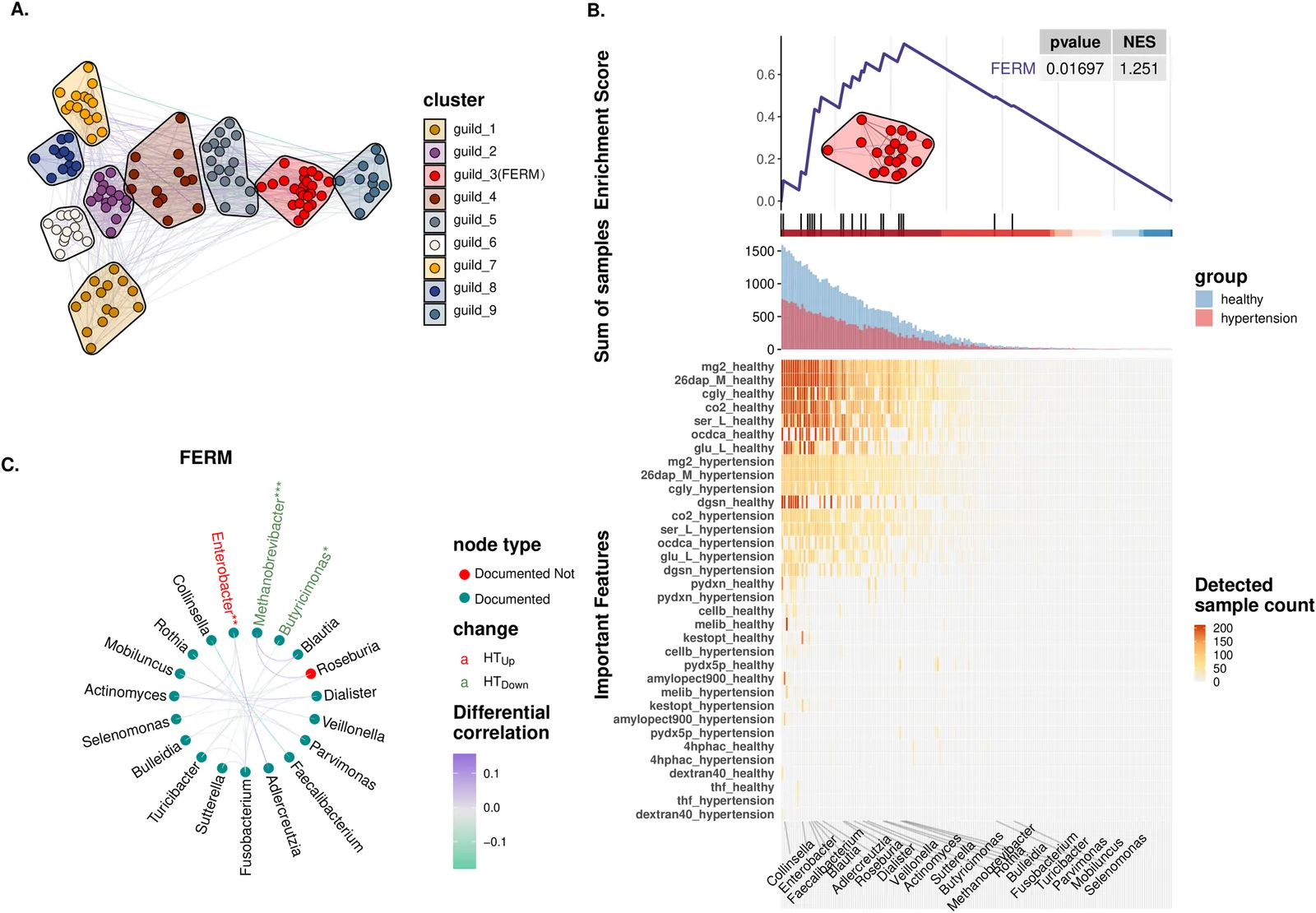
Dysregulation of community-level medium metabolic flux driven by a functional group disturbance: The microbial correlation difference network was divided into nine guilds using the multi-level modularity optimization algorithm (**A**). The GSEA algorithm was used to analyze the enrichment of the 17 key metabolites in microbial guilds. The 17 key metabolites are primarily associated with FERM. NES is the normalized enrichment score. The color of the heatmap below represents the number of samples in which each species is associated with the 17 key metabolites (**B**). The microbial interactions in the FERM have undergone significant changes and a red species in the FERM have already been shown to be associated with hypertension, green edges indicate strengthened negative correlations, and purple edges indicate strengthened positive correlations (**C**).

The multi-level modularity optimization algorithm was employed to cluster nodes in the differential microbial interaction network and identify a total of nine guilds (Fig. 4A). Based on the cross-feeding network, the stability score *Si* was calculated to quantify the associations between microbial species and the 17 key metabolites. Microbial genera were subsequently ranked according to their *Si* values, and microbial guilds were used as candidate gene sets for GSEA to evaluate the enrichment of key metabolite-associated species within each guild. It was found that nodes in the third guild ranked significantly higher in terms of frequency among the important metabolic flux-associated species, with most of the rankings within the top 50 (Fig. 4B; Table S4). In guild 3, there were severe disturbances in the interaction relationships at the genus level, particularly among species such as *Faecalibacterium, Enterobacter, Roseburia*, and *Methanobrevibacter* (FERM). These interactions showed significant differences. From the gutMDisorder database (38), it was found that among species within this guild, *Roseburia* has already been reported to be associated with hypertension. The differential network composed of the FERM guild exhibited significant changes in edge weights (*P* < 0.05), indicating alterations in the strength of interspecies interactions (Fig. 4C). These findings suggest that altered microbial interactions within the FERM guild may contribute to hypertension-associated metabolic disturbances through coordinated effects on key microbial metabolites.

### Significant association between the principal eigenvector of the FERM guild and blood pressure

Additionally, we extracted the principal eigenvector for each functional guild and fitted linear regression models with blood pressure as the outcome. The results showed that the principal eigenvector of FERM was significantly associated with DBP (coefficient = 30.4, *P* = 0.006) and SBP (coefficient = 40.9, *P* = 0.0129). In contrast, the remaining guilds showed few significant associations with blood pressure (Fig. S2A and B; Table S3). In an independent validation cohort of 278 samples, the principal eigenvector of FERM also differed significantly between the hypertensive and healthy groups (Wilcoxon rank-sum test, *P* = 0.0012) (Fig. S2C).

### FERM guild exhibits differential regulatory contributions to key metabolic fluxes

**Fig 5:**
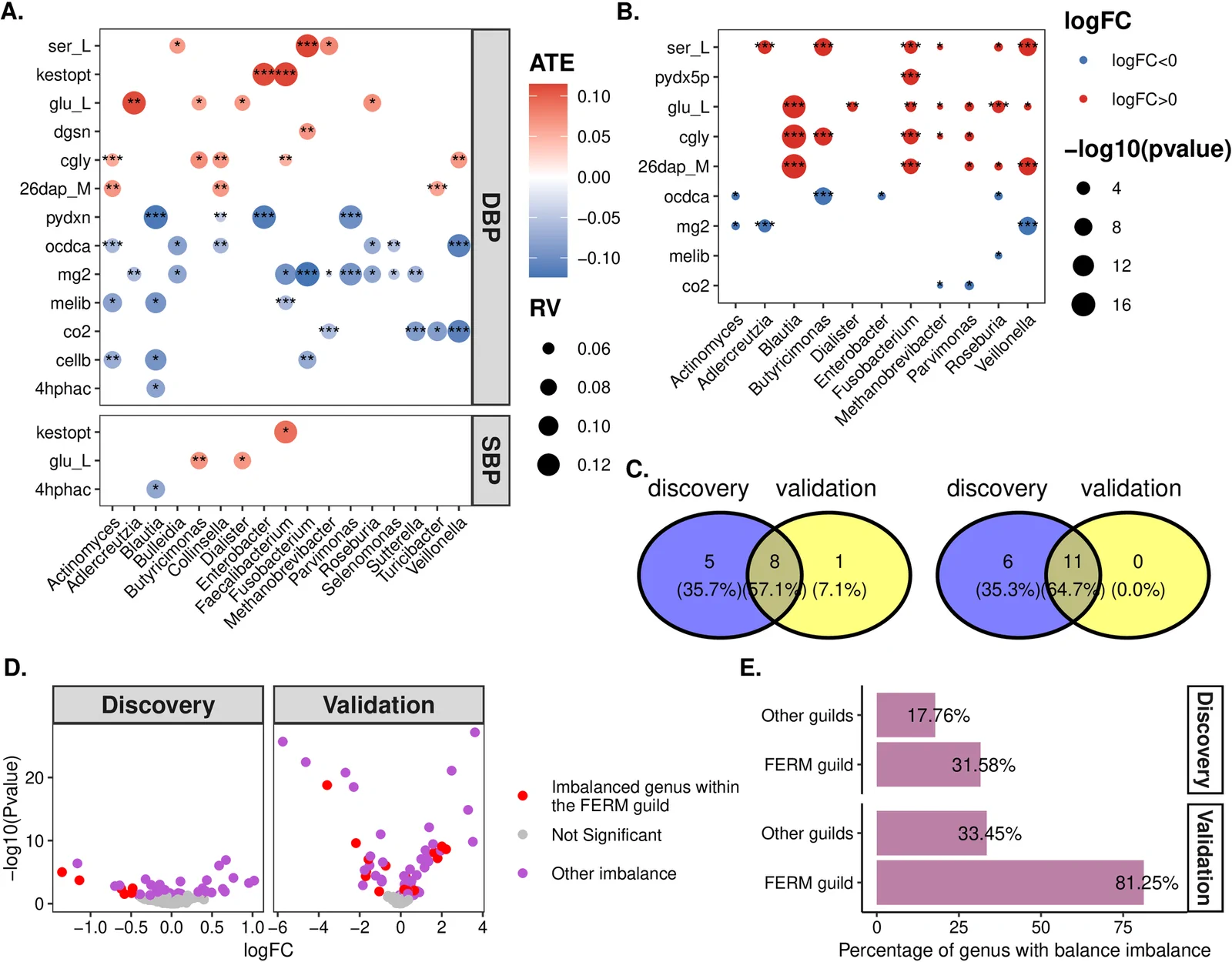
Metabolic flux and interspecies relationship imbalance in the FERM guild: the contribution of FERM genera to key metabolites and its association with blood pressure in the discovery cohort, with red bubbles indicating positive correlation and blue bubbles indicating negative correlation (**A**). The contribution of FERM genera to key metabolites and its association with blood pressure in the validation cohort, with red bubbles indicating positive correlation and blue bubbles indicating negative correlation (**B**). Intersection of key metabolites and FERM genera in the discovery and validation cohorts (**C**). Differential analysis of balance relationships identified by the ADBA distal balance method. Gray indicates no significant difference, red indicates significant differences involving the FERM genera, and blue indicates significant differences involving other genera (**D**). Statistical analysis of balance relationships involving the FERM genera and other genera (**E**).

To assess differential contributions of the FERM guild to key metabolic fluxes, we extracted its associations with key metabolites and applied DoubleML to evaluate their independent effects on blood pressure. In the discovery cohort, 17 FERM species showed significant flux differences involving 13 metabolites, with six positive and seven negative associations with blood pressure (Fig. 5A). In the validation cohort, 11 species and 9 metabolite associations were significant, including five positive and four negative (Fig. 5B). Across cohorts, 11 species-metabolite flux associations showed consistent directional changes between the validation cohort logFC estimates and the ATE signs inferred in the discovery cohort (Fig. 5C), supporting the robustness of these regulatory relationships.

### Pronounced systemic imbalance among FERM species

To comprehensively assess the degree of imbalance within the FERM guild, we used the R package balance to quantify the degree of imbalance between balance pairs of different genera, a method based on log-ratios. In the discovery cohort, we identified 40 significantly imbalanced balances, defined as balance pairs whose log ratio distributions deviated significantly between groups, indicating disrupted relative abundance relationships between the taxa involved .Among these, six of which involved FERM species. In the validation cohort, 63 significant imbalanced balances were detected, including 13 associated with FERM species (Fig. 5D, Table S6). Overall, approximately 31% of FERM species in the discovery cohort exhibited significant imbalance, a proportion that increased to 81% in the validation cohort (Fig. 5E). These results indicate a consistent and widespread functional imbalance of FERM species across independent cohorts.

### Hypertension is associated with increased gut microbial metabolic activity and flux intensification

To investigate the potential impact of gut microbiota dysbiosis on hypertension via microbial cross-feeding mechanisms, we further analyzed the metabolic fluxes of each metabolite by quantifying their production and consumption intensities within the cross-feeding context. After adjusting for potential confounders, we observed that the majority of metabolites in the hypertension group exhibited increased production and uptake fluxes, suggesting an overall enhancement of microbial metabolic activity in hypertensive individuals. (Fig. S3A; Table S7) Furthermore, the adjusted average metabolic fluxes showed no significant correlation with α-diversity but were positively correlated with microbial load. (Fig. S3B) By analyzing the fluxes of microbial substrate consumption and product synthesis, we investigated differences in gut microbial metabolic activity between patients with hypertension and healthy controls. The results revealed significant alterations in both uptake and production fluxes across multiple key metabolic pathways. In particular, pathways related to amino acid metabolism, carbohydrate metabolism, central carbon metabolism, polysaccharide metabolism, and inorganic nutrient metabolism exhibited enhanced activity under hypertensive conditions (Fig. S3C; Table S8). These findings suggest that hypertension is associated with a systematic reprogramming of gut microbial metabolic functions. Notably, enrichment analysis of metabolite production fluxes revealed increased activity of the butyrate metabolism pathway in patients with hypertension. Among the metabolites produced, butyrate showed a non-linear trend with increasing blood pressure, initially rising and then decreasing (Fig. S3D).

## DISCUSSION

This study systematically investigated alterations in gut microbiota composition, metabolic activity, and interspecies interactions under hypertensive conditions. By integrating microbial abundance with community-scale flux modeling, we identified multi-level disruptions in the gut microbial ecosystem, providing evidence for its role in hypertension pathogenesis.

Analysis of α-diversity showed significantly reduced microbial richness (observe, Chao1, and ACE) in hypertensive individuals, while evenness was unchanged. This suggests a loss of unique microbial taxa under hypertensive conditions. Declines in microbial richness are a hallmark of gut dysbiosis and have been widely reported in various metabolic and cardiovascular diseases (39). From a taxonomic perspective, LDA identified multiple differentially abundant genera in the hypertensive group. This trend implies that hypertension may be associated with the loss of potentially protective or regulatory microbial taxa, which may, in turn, impair the functional capacity of the gut microbiota to contribute to host blood pressure homeostasis. β-Diversity analysis showed that the variance explained by the principal coordinates in the PCoA was low, and the $R^{2}$ value from PERMANOVA was only 0.01, suggesting minimal overall structural differences in the gut microbiota between the hypertensive and control groups. These results highlight the need to investigate microbial interactions and metabolic fluxes to better understand hypertension-related dysbiosis.

Investigation of community-level medium metabolites identified 17 metabolites associated with hypertension, many of which have been previously reported to possess pro-inflammatory, anti-inflammatory, antioxidant properties, or function as cofactors for blood pressure-lowering agents. Among them, meso-2,6-diaminoheptanedioate, L-glutamate, and L-cysteinylglycine have demonstrated the potential to directly or indirectly promote hypertension through mechanisms such as inflammation and accumulation of reactive oxygen species. In contrast, magnesium, cellobiose, dextran 40, 5,6,7,8-tetrahydrofolate, amylopectin, melibiose, pyridoxine, 4-hydroxyphenylacetate, and carbon dioxide have been shown to exert antioxidant effects or promote the production of short-chain fatty acids or hypotensive factors. Pyridoxal 5-phosphate and L-serine have exhibited antioxidative or antihypertensive potential in some studies, although other evidence suggests that they may also contribute to elevated blood pressure under certain conditions. Kestopentaose has been reported to enhance short-chain fatty acid synthesis, although a few studies found no significant effect. Octadecanoate (n-C18:0) has predominantly been associated with pro-inflammatory effects, which may be linked to compensatory microbial responses in the gut (Table S9). Notably, the community metabolic modeling framework used in this study was designed to characterize microbiome-associated metabolic capacities rather than to directly predict systemic host metabolic states or circulating metabolite concentrations. Accordingly, the inferred metabolite production and consumption profiles should be interpreted as the potential metabolic contributions of gut microbial communities under defined environmental constraints. We acknowledge that host metabolism, dietary factors, intestinal absorption, and host-microbiome co-metabolism may also substantially influence *in vivo* metabolite levels, which were not explicitly modeled in the current framework. Nevertheless, most existing studies rely on plasma or serum-based metabolomic analyses (40), whereas this study employs a metabolic flux simulation of the gut microbiota to explore microbial metabolic capacity. As such, it offers a novel perspective on the pathophysiological mechanisms of hypertension from the standpoint of microbial functional metabolism.

Although cross-feeding networks showed no differences in node number or density, the hypertension group had shorter path lengths, suggesting more efficient metabolite transfer. Most metabolites exhibited positive effects on blood pressure, and this restructuring was unrelated to α-diversity. This change cannot be simply interpreted as a compensatory mechanism for reduced microbial diversity; species richness is not a key driver of metabolic network restructuring. Instead, this structural alteration is more likely driven by functional redundancy, regulation capacity of key metabolic pathways, or the ecological niche adaptability of certain dominant species, rather than solely relying on overall species count.

Hypertensive individuals’ gut microbiota show enhanced metabolic activity in pathways including methionine/cysteine, alanine/aspartate, the urea cycle, sulfur, and butyrate metabolism, which are central to microbial energy production and linked to host oxidative stress and signaling molecules such as NO and H_2_S (41–43). In individuals with hypertension, the methionine and cysteine metabolic pathway is upregulated, which may affect the levels of homocysteine and H_2_S, thereby influencing vascular function (44). Gut microbiota can convert methionine into homocysteine, and elevated homocysteine levels may promote oxidative stress by inhibiting NO synthesis (45). In contrast, microbiota-derived H_2_S exerts vasodilatory effects (46). Alanine and aspartate can participate in the urea cycle and one-carbon metabolism, providing precursors for NO synthesis (47). In addition, the upregulation of microbial respiratory chain genes enhances the microbiota’s capacity to produce reactive oxygen species (ROS). Trace amounts of ROS or oxidative metabolites may translocate across the intestinal mucosa, inducing low-grade oxidative stress in the host (48, 49). Notably, butyrate metabolism was enhanced in the hypertension group, characterized by an increased production rate of butyrate. As a key short-chain fatty acid, butyrate exhibits multiple physiological functions, including anti-inflammatory effects, vascular protection, and maintenance of intestinal barrier integrity (48, 50). Butyrate flux is initially elevated, possibly as a compensatory response, but declines with progression to grade 2 hypertension, suggesting potential feedback inhibition or metabolic imbalance and warranting further investigation.

Moreover, network module analysis revealed that the disruption of internal interactions within the FERM guild is associated with hypertension. The guild includes *Faecalibacterium, Enterobacter,* and *Roseburia,* which are anaerobic bacteria. Previous studies have demonstrated that Faecalibacterium produces SCFAs, such as butyrate, through the fermentation of dietary fiber (51), while *Enterobacter* is a gram-negative bacterium capable of producing endotoxins such as lipopolysaccharides (LPS), which can trigger host immune responses and lead to inflammation (52). Therefore, *Enterobacter* may contribute to hypertension by producing increased levels of endotoxins, which elicit host immune responses and promote inflammation. *Roseburia* produces butyrate and other SCFAs through the fermentation of complex polysaccharides (53), supporting intestinal barrier integrity and exerting anti-inflammatory effects. Although *Roseburia* has been implicated in hypertension-related studies (54), our study did not observe significant differences in its abundance. However, its interaction patterns with other species were notably altered. Recent studies have shown that even without changes in species abundance, microbial network edge weights can be reorganized in disease, reflecting altered cooperation and competition that affect functional potential (55). Furthermore, using data from two cohorts, we found that the contribution of the FERM genera to key metabolites was significantly associated with blood pressure, and there was also a clear imbalance between the FERM genera and other species.

Based on these findings, we propose the following hypothesis: in hypertension, the gut microbial ecosystem exhibits metabolic dysregulation, characterized primarily by enhanced metabolic activity. This is reflected in the upregulation of pathways such as methionine and cysteine metabolism, the urea cycle, and alanine and aspartate metabolism, which may impair the microbial production of vasoregulatory molecules such as NO and H_2_S, thereby influencing blood pressure regulation. In parallel, the interaction network among gut microbes is substantially altered, particularly within the FERM microbial guild, whose disrupted associations are closely linked to 17 hypertension-related metabolites identified via DoubleML analysis. For example, L-glutamate may modulate blood pressure through neural regulatory mechanisms; 4-hydroxyphenylacetate may promote hypertension by activating pro-inflammatory signaling pathways; fermentation of cellobiose, dextran 40, and amylopectin generates SCFAs, which may influence blood pressure by modulating SCFA synthesis and signaling; and meso-2,6-diaminoheptanedioate may activate NOD1 receptors in intestinal epithelial cells, triggering immune responses that impact vascular function. Additionally, 5,6,7,8-tetrahydrofolate, pyridoxine, and magnesium may participate in blood pressure regulation through their involvement in NO biosynthesis (Fig. S4).

This study proposes several mechanistic hypotheses regarding the relationship between gut microbiota metabolic functions and hypertension, providing potential insights for gut-targeted interventions or therapeutic strategies for hypertension. However, several limitations should be noted. First, the study is based on cross-sectional data, which limits the ability to draw definitive causal inferences. Second, the metabolic flux simulations depend on the accuracy of gut microbial abundance measurements and the completeness of metabolic models in the database. The current modeling framework is based exclusively on microbial genome-scale metabolic models and does not explicitly incorporate host metabolic networks. Therefore, host-related processes, including hepatic metabolism, renal clearance, immune regulation, and dietary absorption, are not directly modeled and may also contribute to the observed circulating metabolite profiles and their associations with blood pressure. In addition, dietary information from the samples was not available; however, since all samples originated from Colombia, Western diet assumptions were uniformly applied as dietary constraints for metabolic flux prediction. Despite these limitations, this study lays a foundation for future mechanistic validation and intervention research on the gut microbiota-hypertension axis. Subsequent studies will integrate longitudinal cohort data, targeted metabolomics, and intervention trials to further validate these key findings and explore the feasibility of modulating the gut microbiome to improve blood pressure regulation.

### Conclusion

This study integrates gut microbial composition, metabolic flux, and microbial interaction networks to reveal structural and functional disturbances of the gut microbiota in hypertension. Hypertensive individuals showed reduced microbial α-diversity, especially in species richness, suggesting a loss of key functional taxa. Differential abundance and LDA analyses confirmed significant community shifts.

Metabolic flux analysis identified 17 metabolites associated with blood pressure regulation, involving inflammation, oxidative stress, and neuroactive pathways. Some metabolites (e.g., meso-2,6-diaminoheptanedioate and L-glutamate) were potentially pro-hypertensive, while others (e.g., magnesium, cellobiose, and tetrahydrofolate) showed antihypertensive and antioxidant effects. Network module analysis revealed that the contribution of the FERM genera to key metabolites is significantly associated with blood pressure, alongside an imbalance between FERM genera and other microbes.

## Data Availability

The species abundance tables used in this study originate from the data set published by Jacobo de la Cuesta-Zuluaga et al., which is accessible at https://github.com/jsescobar/westernization. The data and code used in this study can be accessed in the GitHub repository at https://github.com/as147596/HT.

## SUPPLEMENTAL MATERIAL

Supplemental figures msystems.00358-26-s0001.pdf Figures S1 to S4.

Supplemental text msystems.00358-26-s0002.docx Supplemental methods.

Supplemental tables msystems.00358-26-s0003.xlsx Tables S1-S8.

Table S9 msystems.00358-26-s0004.docx Hypertension-related metabolites identified by DoubleML.

ASM does not own the copyrights to Supplemental Material that may be linked to, or accessed through, an article. The authors have granted ASM a non-exclusive, world-wide license to publish the Supplemental Material files. Please contact the corresponding author directly for reuse.
