# Supplementary material for: The FERM guild: a differentially correlated microbial module drives hypertension via metabolic flux perturbations
Source: mSystems. 2026 Aug 4;11(9):e00358-26. doi: 10.1128/msystems.00358-26 (PMC13595974; doi:10.1128/msystems.00358-26)
Supplement: Supplementary file 1 [file msystems.00358-26-s0001.pdf]

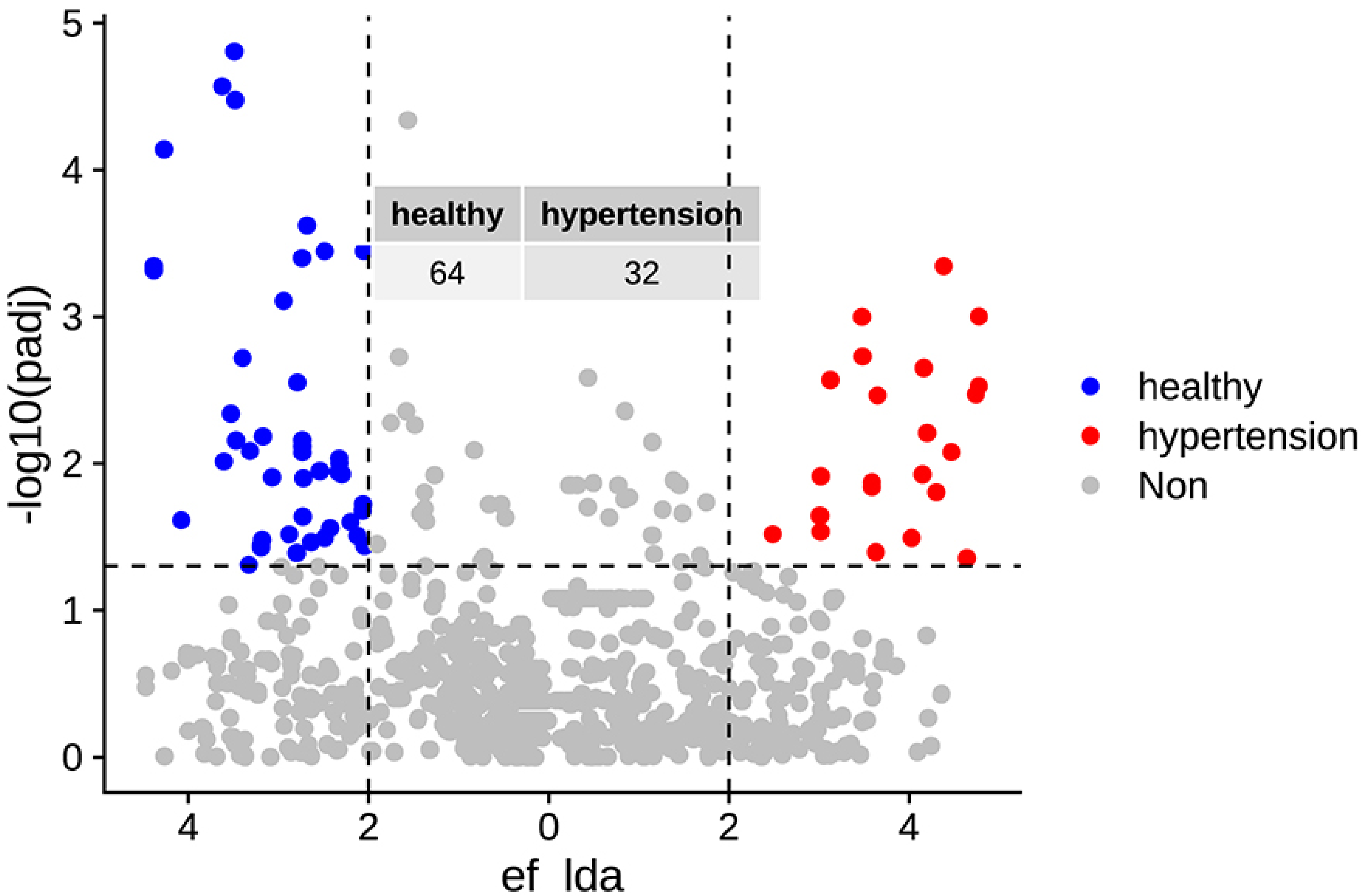

Figure S1. Greater microbial enrichment observed in the healthy group.

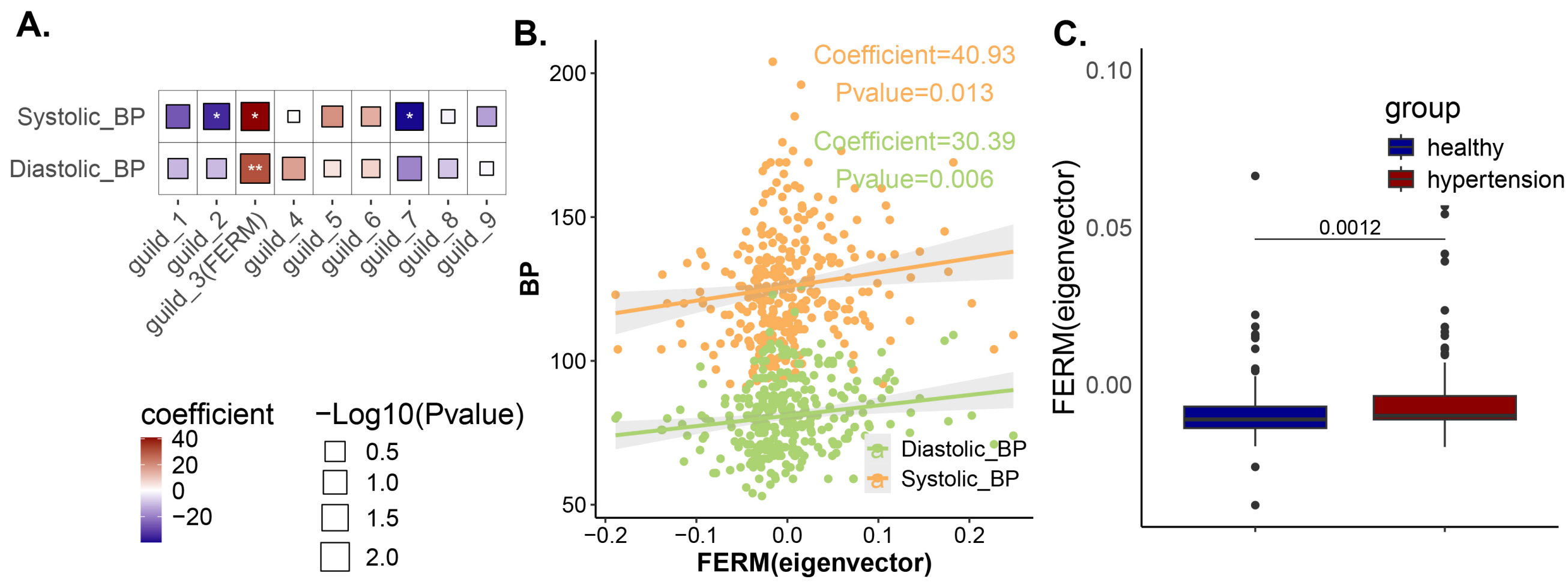

Figure S2. Significant Association Between the Principal Eigenvector of the FERM Guild and Blood Pressure: Linear regression models between guild eigenvectors and blood pressure show that FERM exhibits the strongest associations with both SBP and DBP (A, B). In an independent validation cohort of 278 samples, the FERM eigenvector also differed significantly between the hypertensive and healthy groups, with higher values observed in the hypertensive group (C).

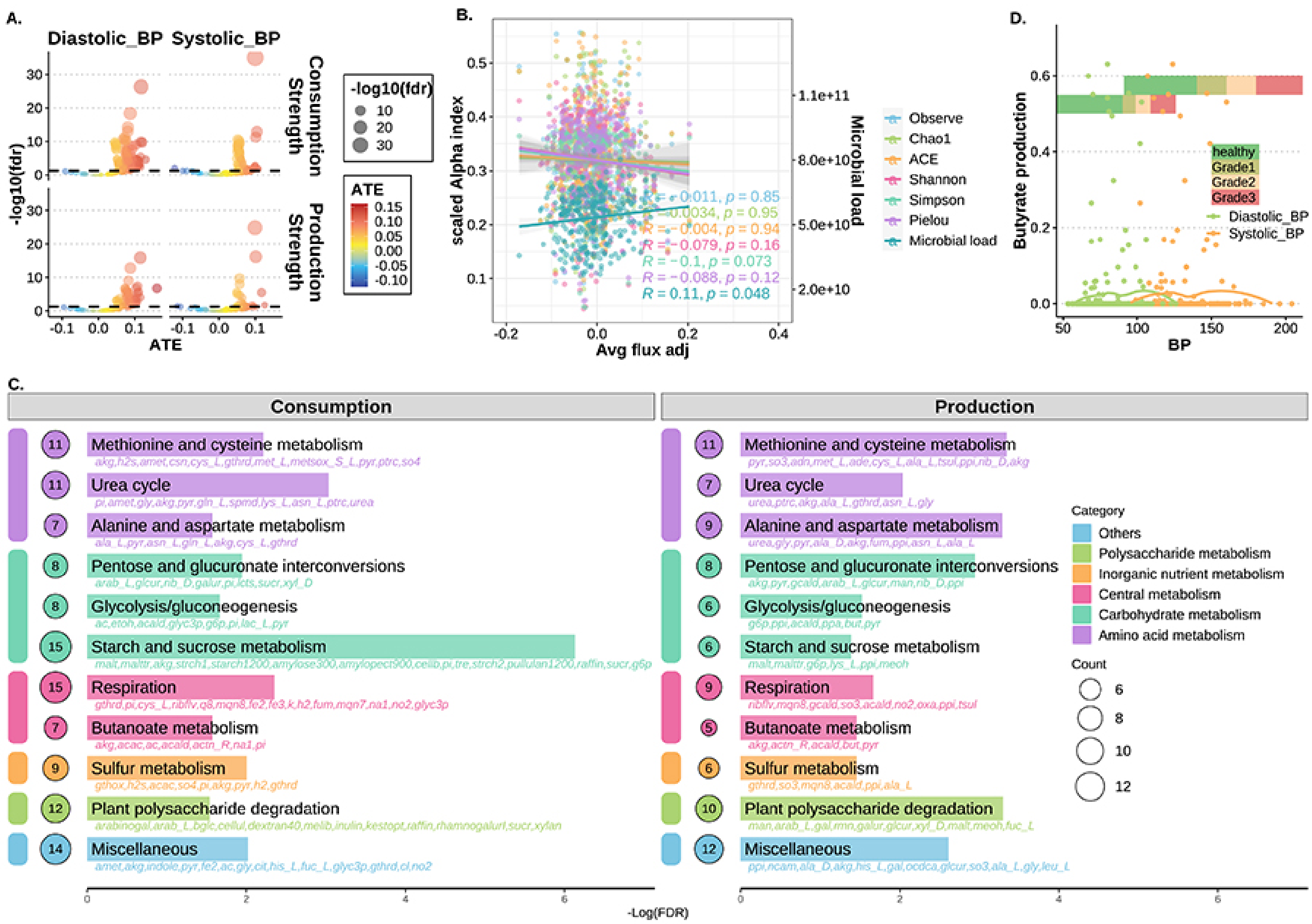

Figure S3. Hypertension Is Associated with Increased Gut Microbial Metabolic Activity and Flux Intensification: Most metabolites exhibit positive average treatment effects on both production and consumption fluxes in relation to blood pressure (A). The average metabolic flux is associated with microbial α-diversity and bacterial load (B). Metabolites with significantly altered production and consumption fluxes are primarily involved in amino acid and carbohydrate metabolism (C). A compensatory metabolic response may be present in individuals with Grade 1 hypertension, but this mechanism appears to diminish in Grade 2 hypertension (D).

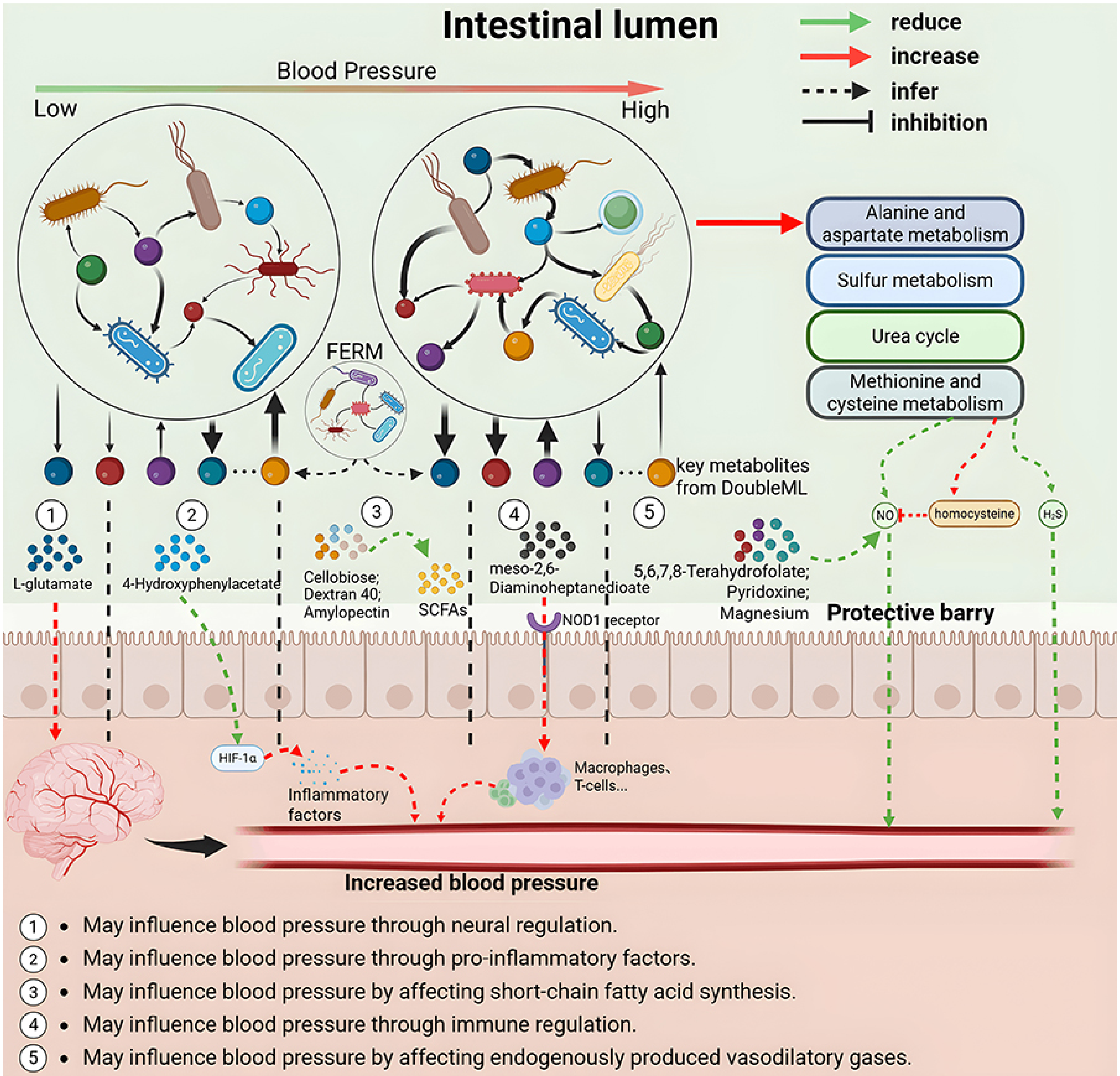

Figure S4. Hypothetical mechanisms of gut microbiota-mediated pathogenesis in hypertension inferred from gut microbiome data mining. The image was created with BioRender.com
