# Supplementary material for: The FERM guild: a differentially correlated microbial module drives hypertension via metabolic flux perturbations
Source: mSystems. 2026 Aug 4;11(9):e00358-26. doi: 10.1128/msystems.00358-26 (PMC13595974; doi:10.1128/msystems.00358-26)
Supplement: Supplementary file 2 [file msystems.00358-26-s0002.docx]

# Supplementary Methods

# Analysis of Gut Microbiota Differences Between Hypertensive and Healthy Populations

## $\alpha$-Diversity Analysis

To explore the relationship between species diversity and hypertension, six $\alpha$-diversity indices of gut microbiota were calculated using the R package MicrobiotaProcess  [1]. These indices reflect the diversity and evenness of species within a habitat or community by calculating the composition and abundance of species within samples [2, 3]. And then the Wilcoxon test was used to compare the significance of microbial alpha diversity between the healthy and hypertensive groups.

## $\beta$-Diversity Analysis

To analyze the overall differences in gut microbiota structure between hypertensive and healthy populations. The PCoA method was used to illustrate the relationships between different groups. First, we calculated the distances between samples using the Bray-Curtis method [4]and evaluated the degree of difference between the two groups using the PERMANOVA method [5].

## Differential Species Analysis

At the genus level, differential abundance analysis was performed using the R package microbiomeMarker [6, 7]. The analysis was carried out with the following parameters: Kruskal–Wallis test cutoff set at 0.01, Wilcoxon test cutoff set to 0.01, LDA score threshold set at 2.

# Cross-Feeding Network Analysis of Gut Microbiota

PFBA was used to infer the metabolic flux profile of each sample, thus quantifying the production and consumption rates of various metabolites by individual microbial species. Based on the resulting flux information, a species–metabolite network was constructed for each sample, where microbial species and metabolites were represented as nodes and metabolic connections between species were established through cross-feeding interactions. To further investigate hypertension-associated metabolic alterations, the weighted in-degree and out-degree of each metabolite within the cross-feeding network were calculated. Subsequently, the Double Machine Learning (DoubleML) framework was applied to estimate the average treatment effect (ATE) of each metabolite on blood pressure (diastolic and systolic) while adjusting for potential confounding variables, including age, adiponectin, BMI, and body fat percentage, and other potential confounders. The Benjamini-Hochberg procedure was used to control the false discovery rate (FDR) in multiple hypothesis testing, and metabolites with FDR-adjusted p-values $<$ 0.05 were identified as significantly associated with blood pressure.

# Fecal Microbial Load Calculation

This study organized and standardized genus-level abundance data of the gut microbiota, and employed the MLP online platform  [18] to build a predictive model for fecal microbial load, thereby obtaining an estimated microbial load value for each sample.

# Correlation Analysis Between Average Metabolic Flux Intensity and Microbial $\alpha$-Diversity

To investigate the relationship between the production and consumption rates of metabolites and microbial community diversity, we calculated the consumption rate $M_{ci}^{\left( k \right)}$ and production rate $M_{pi}^{\left( k \right)}$ of each metabolite in the cross-feeding network of each sample:

$$\begin{matrix} M_{ci}^{\left( k \right)}=\sum_{j}^{m} A_{ij},\left( A_{ij}<0 \right) \end{matrix}$$

$$\begin{matrix} M_{pi}^{\left( k \right)}=\sum_{j}^{m} A_{ij},\left( A_{ij}>0 \right) \end{matrix}$$

Where $k$ denotes the $k$-th sample, $A_{ij}$ represents the consumption intensity of the $j$-th species for the $i$-th metabolite, and m is the number of species capable of producing or consuming this metabolite. Consequently, calculate the average flux intensity $\bar{M}^{\left( k \right)}$ of each metabolite:

$$\begin{matrix} \bar{M}^{\left( k \right)}=\left( M_{ci}^{\left( k \right)}+M_{pi}^{\left( k \right)} \right)/m \end{matrix}$$

Subsequently, we performed confounding adjustment on the average flux intensity$\bar{M}_{adj}^{\left( k \right)}$. Specifically, we constructed a linear model with average flux intensity as the dependent variable and the confounding factors as independent variables. The residuals of this model were extracted and used as the adjusted average flux intensities:

$$\begin{matrix} \bar{M}^{\left( k \right)}=\beta X^{\left( k \right)}+\varepsilon^{\left( k \right)} \end{matrix}$$

$$\begin{matrix} \bar{M}_{adj}^{\left( k \right)}=\varepsilon^{\left( k \right)} \end{matrix}$$

Finally, Spearman rank correlation analysis was performed to evaluate the association between microbial community $\alpha$-diversity and Fecal Microbial Load with the adjusted average flux intensity.

# Pathway Enrichment of Differential Network Nodes

To investigate the metabolic pathways and functions associated with specific metabolites, pathway enrichment analysis was performed. First, metabolites showing significant differences in weighted in-degree (production intensity) or weighted out-degree (consumption intensity) were identified based on the cross-feeding network. Metabolic pathway annotations were obtained from the Virtual Metabolic Human (VMH) database, which catalogs the biochemical reactions involving each metabolite and their corresponding pathways. For metabolites with differential consumption intensity, the set of reactant compounds from reactions assigned to each pathway was used as the metabolite set for enrichment analysis. Similarly, for metabolites with differential production intensity, the set of product compounds from pathway-associated reactions was used for enrichment analysis. Enrichment analysis was performed using the cumulative hypergeometric distribution model for statistical testing, with a significance threshold of FDR $<$ 0.05. Pathways that were significantly enriched in both consumption intensity and production intensity directions were defined as differential pathways.

1.	Xu, S., Zhan, L., Tang, W., Wang, Q., Dai, Z., Zhou, L., Feng, T., Chen, M., Wu, T., Hu, E., others: MicrobiotaProcess: A comprehensive r package for deep mining microbiome. The Innovation. 4, (2023)

2.	Finotello, F., Mastrorilli, E., Di Camillo, B.: Measuring the diversity of the human microbiota with targeted next-generation sequencing. Briefings in bioinformatics. 19, 679–692 (2018)

3.	Chao, A.: Estimating the population size for capture-recapture data with unequal catchability. Biometrics. 783–791 (1987)

4.	Bray, J.R., Curtis, J.T.: An ordination of the upland forest communities of southern wisconsin. Ecological monographs. 27, 326–349 (1957)

5.	Anderson, M.J.: A new method for non-parametric multivariate analysis of variance. Austral ecology. 26, 32–46 (2001)

6.	Cao, Y., Dong, Q., Wang, D., Zhang, P., Liu, Y., Niu, C.: microbiomeMarker: An r/bioconductor package for microbiome marker identification and visualization. Bioinformatics. 38, 4027–4029 (2022)

7.	Segata, N., Izard, J., Waldron, L., Gevers, D., Miropolsky, L., Garrett, W.S., Huttenhower, C.: Metagenomic biomarker discovery and explanation. Genome biology. 12, 1–18 (2011)

8.	Heinken, A., Hertel, J., Acharya, G., Ravcheev, D.A., Nyga, M., Okpala, O.E., Hogan, M., Magnúsdóttir, S., Martinelli, F., Nap, B., others: Genome-scale metabolic reconstruction of 7,302 human microorganisms for personalized medicine. Nature Biotechnology. 41, 1320–1331 (2023)

9.	Noronha, A., Modamio, J., Jarosz, Y., Guerard, E., Sompairac, N., Preciat, G., Danı́elsdóttir, A.D., Krecke, M., Merten, D., Haraldsdóttir, H.S., others: The virtual metabolic human database: Integrating human and gut microbiome metabolism with nutrition and disease. Nucleic acids research. 47, D614–D624 (2019)

10.	Lewis, N.E., Hixson, K.K., Conrad, T.M., Lerman, J.A., Charusanti, P., Polpitiya, A.D., Adkins, J.N., Schramm, G., Purvine, S.O., Lopez-Ferrer, D., others: Omic data from evolved e. Coli are consistent with computed optimal growth from genome-scale models. Molecular systems biology. 6, 390 (2010)

11.	Diener, C., Gibbons, S.M., Resendis-Antonio, O.: MICOM: Metagenome-scale modeling to infer metabolic interactions in the gut microbiota. MSystems. 5, 10–1128 (2020)

12.	Chernozhukov, V., Chetverikov, D., Demirer, M., Duflo, E., Hansen, C., Newey, W., Robins, J.: Double/debiased machine learning for treatment and structural parameters, (2018)

13.	Chernozhukov, V., Cinelli, C., Newey, W., Sharma, A., Syrgkanis, V.: Long story short: Omitted variable bias in causal machine learning. National Bureau of Economic Research (2022)

14.	Gill, R., Datta, S., Datta, S.: A statistical framework for differential network analysis from microarray data. BMC bioinformatics. 11, 1–10 (2010)

15.	Blondel, V.D., Guillaume, J.-L., Lambiotte, R., Lefebvre, E.: Fast unfolding of communities in large networks. Journal of statistical mechanics: theory and experiment. 2008, P10008 (2008)

16.	Subramanian, A., Tamayo, P., Mootha, V.K., Mukherjee, S., Ebert, B.L., Gillette, M.A., Paulovich, A., Pomeroy, S.L., Golub, T.R., Lander, E.S., others: Gene set enrichment analysis: A knowledge-based approach for interpreting genome-wide expression profiles. Proceedings of the National Academy of Sciences. 102, 15545–15550 (2005)

17.	Mootha, V.K., Lindgren, C.M., Eriksson, K.-F., Subramanian, A., Sihag, S., Lehar, J., Puigserver, P., Carlsson, E., Ridderstråle, M., Laurila, E., others: PGC-1$\alpha$-responsive genes involved in oxidative phosphorylation are coordinately downregulated in human diabetes. Nature genetics. 34, 267–273 (2003)

18.	Nishijima, S., Stankevic, E., Aasmets, O., Schmidt, T.S., Nagata, N., Keller, M.I., Ferretti, P., Juel, H.B., Fullam, A., Robbani, S.M., others: Fecal microbial load is a major determinant of gut microbiome variation and a confounder for disease associations. Cell. 188, 222–236 (2025)
