# Supplementary material for: The FERM guild: a differentially correlated microbial module drives hypertension via metabolic flux perturbations
Source: mSystems. 2026 Aug 4;11(9):e00358-26. doi: 10.1128/msystems.00358-26 (PMC13595974; doi:10.1128/msystems.00358-26)
Supplement: Supplementary file 3 [file msystems.00358-26-s0004.docx]

**Table S9.** Hypertension-Related Metabolites Identified by DoubleML (continued)

| **Metabolites^1^** | **ATE^2^** | **Potential mechanisms^3^** | **Summary^4^** |
| --- | --- | --- | --- |
| meso-2,6-Diaminoheptanedioate | positive effect | meso-2,6-Diaminoheptanedioate is a key cross-linking component in the peptidoglycan structure of Gram-negative bacteria and can be recognized by the NOD1 receptor, thereby activating host immune responses. It may serve as a potential biomarker for microbial community shifts and immune stimulation [1]. | Potential to promote hypertension |
| L-glutamate,L-Cysteinylglycine | positive effect | L-cysteinylglycine and L-glutamate are key intermediate metabolites in the catabolism of glutathione (GSH), generated through the hydrolysis of GSH catalyzed by $\gamma$-glutamyl transpeptidase (GGT). Elevated levels of these metabolites typically indicate increased oxidative stress and impaired GSH cycling in the body [2].Glutamate serves not only as the primary excitatory neurotransmitter in the central nervous system but also plays a central role in nitrogen metabolism. Its abnormal expression within the gut–brain axis pathway has been shown to influence blood pressure homeostasis via autonomic nervous system regulation and inflammatory signaling pathways [3].L-cysteinylglycine can promote reactive oxygen species (ROS) generation and deplete nitric oxide ($NO$) reserves, thereby impairing vascular endothelial function [4]. | Potential to promote hypertension |
| Carbon dioxide | Negative effect | Elevated $CO_{2}$ levels in the systemic circulation (hypercapnia) can induce vasodilation through the release of endothelial $NO$. For example, cerebral blood vessels are highly sensitive to $CO_{2}$; increased $CO_{2}$ concentration enhances local $NO$ production, resulting in a significant rise in blood flow [5, 6]. Although local$CO_{2}$ production in the colon contributes minimally to systemic $CO_{2}$ levels, a reduction in $CO_{2}$ generation may slightly diminish NO-mediated vasodilatory signaling. | Potential to alleviate hypertension |
| Magnesium | Negative effect | Magnesium is a natural calcium channel blocker that reduces intracellular calcium and sodium levels while increasing potassium levels, thereby relaxing smooth muscle and lowering vascular resistance. Additionally, magnesium enhances $NO$ production and improves endothelial function [7, 8]. Studies have shown that daily supplementation with 500–1000 mg of magnesium can lower systolic and diastolic blood pressure by approximately 5.6/2.8 mmHg on average [9]. | Potential to alleviate hypertension |
| Cellobiose | Negative effect | Cellobiose is a major component of cellulose, consisting of two glucose units linked by a $\beta$-1,4 glycosidic bond. Since the human small intestine lacks the enzymes to degrade it, cellobiose passes directly into the colon, where it is fermented by gut microbiota. This fermentation produces large amounts of SCFAs, such as acetate, propionate, and butyrate, as well as small amounts of hydrogen, methane, and carbon dioxide [10]. These SCFAs can transiently lower blood pressure by activating host receptors such as GPR41 and GPR43, which induce vasodilation and suppress inflammation [11]. | Potential to alleviate hypertension |
| Dextran 40 | Negative effect | Dextran can promote the growth of beneficial gut bacteria and is fermented by colonic microbiota to produce SCFAs [12, 13]. | Potential to alleviate hypertension |
| 5,6,7,8-Tetrahydrofolate | Negative effect | 5,6,7,8-Tetrahydrofolate is the biologically active form of folate. Meta-analyses have shown that daily high-dose folic acid supplementation (approximately 5 mg) for more than six weeks can lead to a slight reduction in systolic blood pressure (around 2 mmHg) in patients with hypertension, along with a significant improvement in endothelial-dependent vasodilation [14]. | Potential to alleviate hypertension |
| Amylopectin | Negative effect | Amylopectin is fermented in the colon by gut bacteria such as Roseburia spp. and Eubacterium rectale, leading to the production of SCFAs [11, 15]. | Potential to alleviate hypertension |
| Melibiose | Negative effect | As a prebiotic, melibiose can selectively promote the proliferation of beneficial bacteria, particularly Bifidobacterium species, whose metabolic products include acetate and lactate [16]. | Potential to alleviate hypertension |
| Pyridoxine | Negative effect | After host absorption, pyridoxine is primarily converted in the liver into the active coenzyme pyridoxal phosphate. Pyridoxal phosphate regulates intracellular calcium ion transport by inhibiting voltage-gated and ATP-mediated calcium channels, thereby reducing calcium influx in vascular smooth muscle cells [17]. | Potential to alleviate hypertension |
| 4-hydroxyphenylacetate | Negative effect | 4-Hydroxyphenylacetic acid (4-HPA) exhibits anti-inflammatory and antioxidant properties. Studies have shown that 4-HPA can inhibit the expression of $HIF-1\alpha$, thereby reducing the release of pro-inflammatory cytokines and vascular permeability [18]. Additionally, 4-HPA promotes the nuclear translocation of Nrf2 and upregulates the expression of antioxidant enzymes such as GCLC, enhancing cellular antioxidant capacity [19]. | Potential to alleviate hypertension |
| Pyridoxal 5-phosphate | positive effect | Pyridoxal 5-phosphate (PLP), the active form of vitamin B6, serves as a coenzyme for various enzymatic reactions. In hypertensive rat models, PLP has been shown to facilitate the conversion of the vasoconstrictor peptide angiotensin II (Ang II) into a less active derivative (Ang P), thereby reducing calcium influx into vascular smooth muscle cells and attenuating renal vasoconstriction [20]. Additionally, studies have demonstrated that aromatic amino acid decarboxylases in gut microbiota utilize PLP as a cofactor to convert tyrosine into tyramine. Tyramine, a vasoactive amine, can induce potent hypertensive effects through activation of the sympathetic nervous system and trace amine-associated receptors (TAARs) [21, 22]. | Controversial association with blood pressure |
| L-Serine | positive effect | L-serine is a non-essential amino acid that has been shown in animal experiments to exert a significant antihypertensive effect. In rat models, intravenous administration of L-serine induces a marked reduction in blood pressure, an effect that is associated with the activation of calmodulin-dependent potassium channels ($K\_Ca$) [23]. However, other studies suggest that L-serine also serves as a precursor for glycine and D-serine, the latter of which acts as a co-agonist of the N-methyl-D-aspartate receptor (NMDAR). This activity may enhance sympathetic nervous system activity and promote catecholamine release, leading to vasoconstriction [24, 25]. | Controversial association with blood pressure |
| Kestopentaose | positive effect | Kestopentaose is a type of inulin-type fructooligosaccharide (FOS) composed of five monosaccharide units (four fructose and one glucose residues), commonly used as a prebiotic to modulate gut microbiota. Additionally, FOSs have been reported to improve blood lipid profiles and body weight, which may indirectly alleviate vascular stiffness and lower blood pressure [26]. However, clinical trial results remain inconsistent. Systematic reviews suggest that inulin-type FOSs are beneficial for lipid metabolism and weight management, but the evidence supporting a direct blood pressure-lowering effect is limited [27]. | Controversial association with blood pressure |
| Deoxyguanosine | positive effect | Deoxyguanosine itself is a nucleoside component of DNA, but its oxidized product, 8-hydroxy-2’-deoxyguanosine (8-OHdG), has been found at significantly elevated levels in the plasma or urine of hypertensive patients, indicating increased oxidative stress-induced damage [28]. | Biomarker of oxidative stress-induced damage |
| octadecanoate(n-C18:0) | Negative effect | A diet high in saturated fats is commonly associated with an increased risk of atherosclerosis and hypertension [29]. Stearic acid (a saturated fatty acid) can activate pathways such as TLR4 in the body, promoting the release of pro-inflammatory cytokines, and may also affect vascular reactivity by altering membrane lipid composition [30]. | Potential to promote hypertension |

^1^Metabolites identified as associated with hypertension.

^2^Average Treatment Effect estimated by DoubleML.

^3^Mechanisms through which the metabolite may influence blood pressure, based on prior studies.

^4^Summary of the metabolite’s effect direction on hypertension.

1.	Girardin, S.E., Boneca, I.G., Carneiro, L.A., Antignac, A., Jehanno, M., Viala, J., Tedin, K., Taha, M.-K., Labigne, A., Zäthringer, U., others: Nod1 detects a unique muropeptide from gram-negative bacterial peptidoglycan. Science. 300, 1584–1587 (2003)

2.	Akakpo, J.Y.: The protective potential of 4-methylpyrazole (4MP) against acetaminophen (APAP) overdose, (2021)

3.	Yang, T., Zubcevic, J.: Gut–brain axis in regulation of blood pressure. Frontiers in physiology. 8, 845 (2017)

4.	Mendis, S., Athauda, S., Naser, M., Takahashi, K.: Association between hyperhomocysteinaemia and hypertension in sri lankans. Journal of international medical research. 27, 38–44 (1999)

5.	Iadecola, C.: Does nitric oxide mediate the increases in cerebral blood flow elicited by hypercapnia? Proceedings of the National Academy of Sciences. 89, 3913–3916 (1992)

6.	Toda, N., Ayajiki, K., Okamura, T.: Cerebral blood flow regulation by nitric oxide: Recent advances. Pharmacological reviews. 61, 62–97 (2009)

7.	Touyz, R.: Role of magnesium in the pathogenesis of hypertension. Molecular aspects of medicine. 24, 107–136 (2003)

8.	Rosanoff, A., Weaver, C.M., Rude, R.K.: Suboptimal magnesium status in the united states: Are the health consequences underestimated? Nutrition reviews. 70, 153–164 (2012)

9.	Houston, M.: The role of magnesium in hypertension and cardiovascular disease. The Journal of Clinical Hypertension. 13, 843–847 (2011)

10.	Moré, M.I., Postrach, E., Bothe, G., Heinritz, S., Uebelhack, R.: A dose-escalation study demonstrates the safety and tolerability of cellobiose in healthy subjects. Nutrients. 12, 64 (2019)

11.	Poll, B.G., Cheema, M.U., Pluznick, J.L.: Gut microbial metabolites and blood pressure regulation: Focus on SCFAs and TMAO. Physiology. 35, 275–284 (2020)

12.	Koyama, Y., Miyagawa, T., Kawaide, A., Kataoka, K.: Receptor-mediated absorption of high molecular weight dextrans from intestinal tract. Journal of controlled release. 41, 171–176 (1996)

13.	Gościniak, A., Lainé, E., Cielecka-Piontek, J.: How do cyclodextrins and dextrans affect the gut microbiome? Review of prebiotic activity. Molecules. 29, 5316 (2024)

14.	McRae, M.P.: High-dose folic acid supplementation effects on endothelial function and blood pressure in hypertensive patients: A meta-analysis of randomized controlled clinical trials. Journal of chiropractic medicine. 8, 15–24 (2009)

15.	Flint, H.J., Scott, K.P., Duncan, S.H., Louis, P., Forano, E.: Microbial degradation of complex carbohydrates in the gut. Gut microbes. 3, 289–306 (2012)

16.	Adamberg, K., Adamberg, S., Ernits, K., Larionova, A., Voor, T., Jaagura, M., Visnapuu, T., Alamäe, T.: Composition and metabolism of fecal microbiota from normal and overweight children are differentially affected by melibiose, raffinose and raffinose-derived fructans. Anaerobe. 52, 100–110 (2018)

17.	Dakshinamurti, S., Dakshinamurti, K.: Antihypertensive and neuroprotective actions of pyridoxine and its derivatives. Canadian journal of physiology and pharmacology. 93, 1083–1090 (2015)

18.	Liu, Z., Xi, R., Zhang, Z., Li, W., Liu, Y., Jin, F., Wang, X.: 4-hydroxyphenylacetic acid attenuated inflammation and edema via suppressing HIF-1$\alpha$ in seawater aspiration-induced lung injury in rats. International Journal of Molecular Sciences. 15, 12861–12884 (2014)

19.	Zhao, H., Jiang, Z., Chang, X., Xue, H., Yahefu, W., Zhang, X.: 4-hydroxyphenylacetic acid prevents acute APAP-induced liver injury by increasing phase II and antioxidant enzymes in mice. Frontiers in pharmacology. 9, 653 (2018)

20.	Lellig, M., Rodrı́guez, M., López-Baltanás, R., Hermann, J., Wollenhaupt, J., Noels, H., Zidek, W., Tepel, M., Mahfoud, F., Jankowski, J., others: Pyridoxal-5′-phosphate: A cost-effective treatment candidate for hypertensive patients? Journal of internal medicine. 296, 435–448 (2024)

21.	Aydin, S., Ugur, K., Aydin, S.: Could excessive production of tyramine by the microbiota be a reason for essential hypertension? Bioscience of Microbiota, Food and Health. 37, 77–78 (2018)

22.	Battersby, E.J., Newman, E.V.: Chronotropic effects of norepinephrine and tyramine: A system response analysis. Circulation Research. 15, 112–123 (1964)

23.	Mishra, R.C., Tripathy, S., Quest, D., Desai, K.M., Akhtar, J., Dattani, I.D., Gopalakrishnan, V.: L-serine lowers while glycine increases blood pressure in chronic l-NAME-treated and spontaneously hypertensive rats. Journal of hypertension. 26, 2339–2348 (2008)

24.	Suwandhi, L., Hausmann, S., Braun, A., Gruber, T., Heinzmann, S.S., Gálvez, E.J., Buck, A., Legutko, B., Israel, A., Feuchtinger, A., others: Chronic d-serine supplementation impairs insulin secretion. Molecular metabolism. 16, 191–202 (2018)

25.	Wolosker, H., Balu, D.T.: D-serine as the gatekeeper of NMDA receptor activity: Implications for the pharmacologic management of anxiety disorders. Translational psychiatry. 10, 184 (2020)

26.	Yeo, S.-K., Ooi, L.-G., Lim, T.-J., Liong, M.-T.: Antihypertensive properties of plant-based prebiotics. International journal of molecular sciences. 10, 3517–3530 (2009)

27.	Talukdar, J.R., Cooper, M., Lyutvyn, L., Zeraatkar, D., Ali, R., Berbrier, R., Janes, S., Ha, V., Darling, P.B., Xue, M., others: The effects of inulin-type fructans on cardiovascular disease risk factors: Systematic review and meta-analysis of randomized controlled trials. The American Journal of Clinical Nutrition. 119, 496–510 (2024)

28.	Di Minno, A., Turnu, L., Porro, B., Squellerio, I., Cavalca, V., Tremoli, E., Di Minno, M.N.D.: 8-hydroxy-2-deoxyguanosine levels and cardiovascular disease: A systematic review and meta-analysis of the literature. Antioxidants & redox signaling. 24, 548–555 (2016)

29.	Kochumon, S., Arefanian, H., Azim, R., Shenouda, S., Jacob, T., Abu Khalaf, N., Al-Rashed, F., Hasan, A., Sindhu, S., Al-Mulla, F., others: Stearic acid and tnf-$\alpha$ co-operatively potentiate mip-1$\alpha$ production in monocytic cells via myd88 independent tlr4/tbk/irf3 signaling pathway. Biomedicines. 8, 403 (2020)

30.	Maki, K.C., Dicklin, M.R., Kirkpatrick, C.F.: Saturated fats and cardiovascular health: Current evidence and controversies, (2021)
